# Ocular Nystagmus as the Initial Presenting Feature in a Patient with Complete *CLTC* Deletion: Expanding the Genotype–Phenotype Spectrum of *CLTC*-Related Disorder

**DOI:** 10.3390/genes17080928

**Published:** 2026-08-08

**Authors:** Xin Yang, Rongrong Pan, Fan Yu, Huidan Wu, Neil Manish Shah, Hong Li

**Affiliations:** 1 Department of Genetics and Metabolism, Children’s Hospital, Zhejiang University School of Medicine, National Clinical Research Center for Children and Adolescents’ Health and Diseases, Hangzhou 310052, China; 3190104275@zju.edu.cn (R.P.); 22518728@zju.edu.cn (F.Y.); wuhuidan@zju.edu.cn (H.W.); 2 Zhejiang Key Laboratory of Neonatal Diseases, Hangzhou 310052, China; 3 Binjiang Institute of Zhejiang University, Hangzhou 310053, China; 4 Department of Human Genetics, Emory University School of Medicine, Atlanta, GA 30322, USA; neil.shah@emoryhealthcare.org

**Keywords:** *CLTC*-related disorder, neurodevelopmental disorder, congenital nystagmus, 17q23.1 microdeletion

## Abstract

**Background**: *CLTC*-related neurodevelopmental disorder is a rare condition primarily characterized by global developmental delay (GDD) and intellectual disability (ID). To date, approximately 41 cases involving *CLTC* gene alterations have been reported. We present the first individual with a complete deletion of the *CLTC* gene. **Methods:** The proband is a male from a non-consanguineous family, presenting with congenital nystagmus, hypotonia, GDD, and autism spectrum disorder (ASD). Chromosomal microarray analysis and trio exome sequencing were performed. A systematic review of previously reported *CLTC* variant cases was conducted to delineate the phenotypic spectrum. **Results:** A *de novo* 363-kb heterozygous deletion at 17q23.1 spanning the entire *CLTC* gene was identified. The systematic review confirmed GDD/ID as core features and revealed various ocular abnormalities in a subset of cases. These findings indicate that the clinical phenotype extends beyond neurodevelopment, with multi-system involvement. **Conclusions:** The phenotypic heterogeneity of *CLTC*-related disorders underscores the need for comprehensive physical examination to identify extra-neurological manifestations. Accurate diagnosis relies on integrating detailed clinical phenotyping with comprehensive genomic testing. Early, precise diagnosis facilitates multidisciplinary management, informed genetic counseling, and the establishment of long-term surveillance protocols.

## 1. Introduction

Clathrin is a pivotal scaffold protein that assembles into characteristic triskelion-shaped structures, each composed of three clathrin heavy chains (CHCs) and three light chains. These specialized complexes are instrumental in intracellular trafficking, particularly in mediating the endocytosis of receptors and various macromolecules [1,2,3,4]. Beyond its classical role in vesicle formation, clathrin is integral to mitotic spindle function. As a key component of the TACC3/ch-TOG/clathrin complex, it facilitates the stabilization of kinetochore fibers by forming inter-microtubule bridges, which are essential for maintaining spindle tension during mitosis [5,6]. Furthermore, clathrin participates in several other critical physiological processes, including synaptic vesicle recycling, hormone desensitization, and spermiogenesis [1,7]. Its remarkable conformational flexibility allows it to adopt various lattice structures, enabling its functional versatility within different cellular contexts.

CHC serves as the primary structural scaffold of the clathrin triskelion and is fundamental to the molecular mechanisms governing clathrin-mediated pathways [8,9]. Within the nervous system, CHC is indispensable for synaptic vesicle recycling and neurotransmitter release at presynaptic terminals [10]. Furthermore, CHC deficiency has been linked to various mitotic aberrations, such as spindle assembly defects, kinetochore fibers instability, and protracted mitosis resulting from persistent spindle assembly checkpoint signaling [5].

The CHC protein is encoded by *CLTC* gene (OMIM 118955), located on human chromosome 17q23.1 and highly expressed throughout the brain [11]. Since DeMari et al. first identified *CLTC* as a disease-causing gene for multiple malformations and developmental delay in 2016 [11], pathogenic variants in this gene have been reported in sparse case series. These variants are associated with a broad neurodevelopmental spectrum, encompassing global developmental delay (GDD), intellectual disability (ID), epilepsy, movement disorders, and others [12,13,14]. Owing to the extreme rarity of this condition, the full clinical landscape and the precise genotype–phenotype correlations remain to be fully elucidated.

In this study, we report a novel and unique case characterized by a complete *CLTC* gene deletion to date. In addition to the characteristic neurodevelopmental phenotypes, this patient exhibited congenital nystagmus and limited extension of the upper limbs, potentially expanding the phenotypic spectrum of the disorder. To further contextualize these findings, we comprehensively reviewed and analyzed clinical and genetic data from 42 individuals to better delineate genotype–phenotype correlations in this rare condition, thereby informing genetic counseling and surveillance strategies.

## 2. Materials and Methods

### 2.1. Patient Evaluation

A 14-year-old male initially presented at birth with severe congenital nystagmus, subsequently manifesting GDD, hypotonia, and autism spectrum disorder (ASD) was evaluated for a possible genetic etiology by a child psychiatrist and a clinical geneticist. Clinical findings, developmental history, pedigree, and physical examination were comprehensively reviewed at Emory University Hospital. Informed consent was obtained from his legal guardians.

### 2.2. Genetic Analysis

Genomic DNA was extracted from peripheral blood. High-resolution chromosomal microarray analysis (CMA) was performed to detect copy number variations (CNVs), with data analyzed relative to the human reference genome GRCh37 (hg19). The clinical relevance of the identified CNVs was evaluated by cross-referencing public genomic databases, including DGV, DECIPHER, and OMIM. Whole-exome sequencing (WES) was conducted on a high-throughput platform with a target mean coverage of ≥100×. Bioinformatic analysis included sequence alignment to the reference genome, variant calling, and functional annotation. Variant interpretation followed the American College of Medical Genetics and Genomics (ACMG) guidelines. The deletion was confirmed as *de novo* through trio-based analysis incorporating parental samples.

### 2.3. Systematic Literature Review and Data Synthesis

To delineate the genotype–phenotype landscape of *CLTC*-related disorders, a systematic search of PubMed was conducted for peer-reviewed publications up to March 2026. The search strategy employed the following terms: “*CLTC*”, “*CLTC* variant” and “*CLTC*-related disease”. Eligible studies included pediatric and adult case reports, case series, and detailed clinical reviews published in English. Cases harboring *CLTC* variants but lacking sufficient phenotypic data were excluded. To ensure robust synthesis of existing evidence, clinical findings from the present case were integrated with those from previously reported cases [11,13,15,16,17,18,19,20,21,22,23,24,25,26]. A detailed summary is available in Table S1.

## 3. Results

### 3.1. Case Presentation

The patient is a 14-year-old male with congenital nystagmus, hypotonia, GDD and ASD. He was born at 37 weeks’ gestation (birth weight 3770 g) to a 23-year-old mother via uncomplicated delivery. No neonatal intensive care was required.

At birth, significant nystagmus was observed, prompting a brain MRI that revealed mild hydrocephalus which was monitored without intervention. A concurrent ophthalmic examination showed no structural or retinal abnormalities. During early childhood, gross motor milestones were significantly delayed. The patient had hypotonia and achieved independent sitting at 13 months, pulling to stand at 24 months, and independent walking at 3 years of age. Language development was similarly delayed, with his first meaningful words occurring at age 3, and he can only speak short sentences currently. Academically, he exhibits a mild learning disability and follows an Individualized Education Program (IEP), performing at a third-grade level in mathematics. Notably, he faces significant challenges with writing, whereas his reading abilities are relatively preserved. Behaviorally, the patient exhibits echolalia, occasionally temper tantrums, and hypersensitivity to loud noises. Due to these features alongside repetitive and stereotyped behaviors, he was diagnosed with ASD at age 5.

His medical history is notable for nocturnal enuresis, asthma, atopic dermatitis, and sensitivities to lactose and gluten. He has experienced chronic gastrointestinal issues, including feeding difficulties, gastroesophageal reflux, abdominal pain, vomiting, diarrhea, and constipation. At age 5, he underwent surgical repair of a left inguinal hernia. He has no history of seizures. Serial brain MRIs at ages 6 and 10 demonstrated resolution of the hydrocephalus and identified stable prominent draining vein in the right occipital lobe and the LEFT temporal lobe with normal brain parenchymal signal and morphology.

Physical examination revealed growth parameters within normal limits, with body mass index (BMI) below the 5th percentile. No facial dysmorphism was observed. Nystagmus was persistently present and became more pronounced when the contralateral eye was covered. Additionally, bilateral elbow joint contractures limited upper limb extension, preventing full straightening of the arms and resulting in a slightly abnormal gait.

CMA identified a *de novo* 363-kb heterozygous deletion of 17q23.1 (arr [GRCh37]17q23.1(57624793-57988073) x1), encompassing *CLTC*, *PTRH2*, *DHX40*, *VMP1*, *MIR21* and *TUBD1*. Among these, only *CLTC* and *PTRH2* (OMIM:608625) are associated with known disorders. However, *PTRH2*-related multisystem disease is autosomal recessive, inconsistent with our findings. Given that heterozygous *CLTC* variants are linked to neurodevelopmental disorders, this deletion was considered the primary candidate for the patient’s phenotypes. Trio exome sequencing identified no additional pathogenic variants in genes associated with congenital nystagmus, further supporting *CLTC* haploinsufficiency as the cause of the ocular phenotype.

### 3.2. Literature Review

To date, a total of 42 individuals with variants in the *CLTC* gene have been documented, including the new patient first reported in this study above (22 females and 20 males). The age at the time of clinical evaluation ranged from 3 months to 41 years (Table 1 and Table S1). With the exception of one neonatal death, all patients were alive at the time of publication.

### 3.3. Phenotype of CLTC-Related Disorder

**Figure 1:**
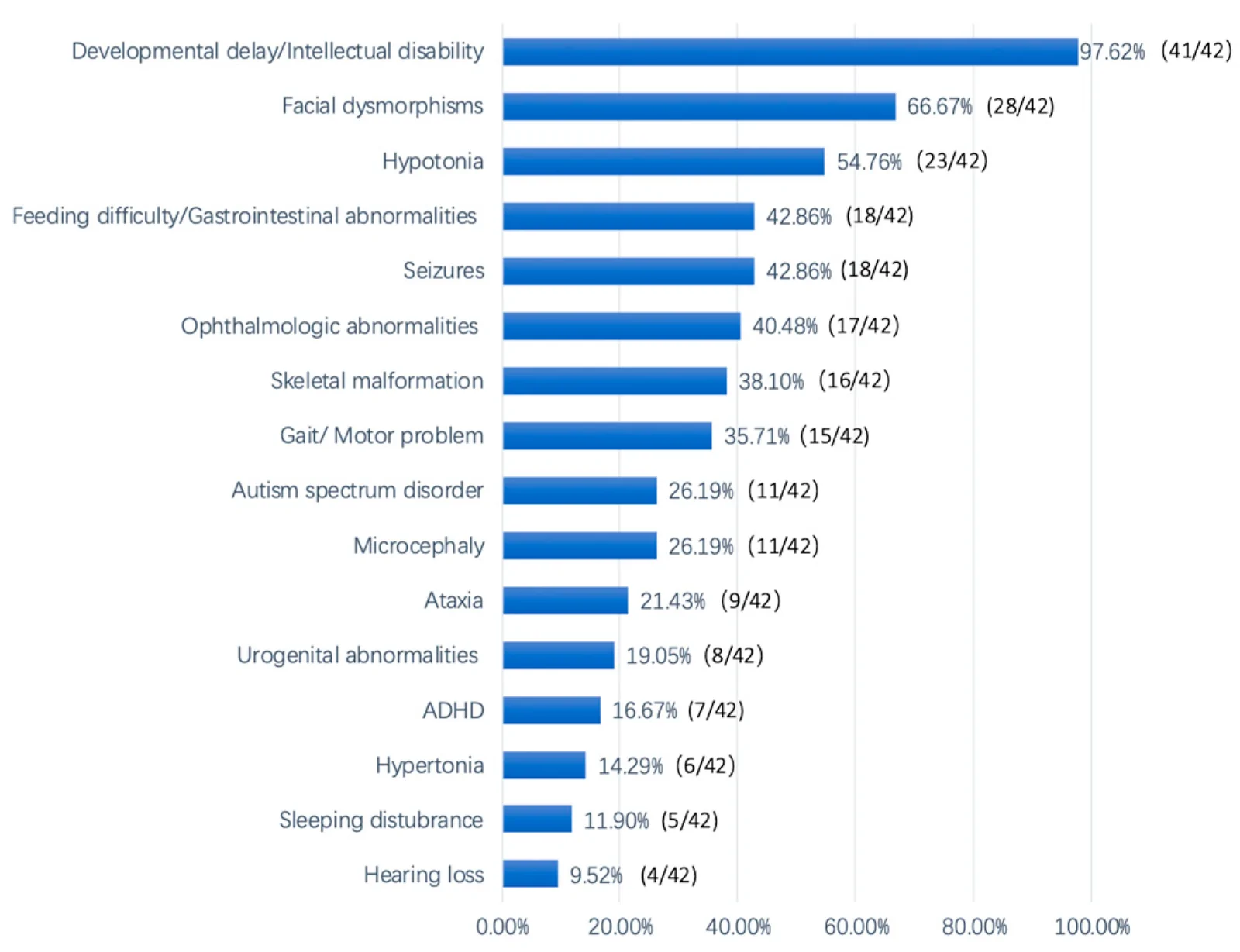
Distribution of various clinical features.

Alterations in the *CLTC* gene manifest as a multisystem disorder, primarily characterized by neurological symptoms (including developmental delay, seizures, and abnormal muscle tone), congenital malformation, gastrointestinal dysfunction, alongside ocular, urogenital, auditory, and sleep-related complications (Figure 1, Table S1).

Except for one individual with a mosaic variant who exhibited normal development, the rest of individuals with *CLTC*-related disorder exhibited developmental delay (DD) and/or ID (41/42). Among them, 30 individuals exhibited mild-to-severe GDD, two presented with isolated speech delay, five with isolated motor delay. Notably, ID is also the most prevalent symptom. Excluding six patients with insufficient medical history for ID assessment, the other 35 patients presented with ID of different severities.

Other neurological manifestations were remarkably diverse. Abnormal muscle tone was frequently observed in early childhood, with hypotonia being more common (23/42) than hypertonia (6/42). These motor impairments often culminated in gait abnormalities and balance deficits (15/42). Seizures were reported in 18 patients (18/42), with onset ranging from the neonatal period to adulthood; notably, most were well-controlled with pharmacotherapy. In patients with seizures, nearly all displayed concurrent abnormalities on brain MRI or EEG. Overall, neuroimaging revealed structural brain abnormalities in approximately two-thirds of the cohort (23/34). Other observed features included microcephaly (11/42), ataxia (9/42), and behavioral issues such as ASD (11/42), attention deficit hyperactivity disorder (ADHD) (7/42), and emotional disturbances. Rare neurological signs included bradykinesia, oral–motor apraxia, and myoclonus.

In addition to neurological symptoms, individuals with *CLTC*-related disorder also present with manifestations involving other systems. Gastrointestinal issues represent the most frequent non-neurological findings (18/42), predominantly manifesting as gastroesophageal reflux and feeding difficulties. Ophthalmological abnormalities were observed in nearly half of the cohort (17/42), including strabismus, nystagmus, congenital ptosis, and visual impairment. Regarding the persistent nystagmus observed in our patient, our review identified three other female patients with similar symptom, one of whom presented as early as six hours postpartum.

Furthermore, approximately two-thirds of the patients (28/42) exhibited distinctive craniofacial dysmorphism, such as a high-arched palate, long philtrum, low-set ears, epicanthus, and a depressed nasal bridge. Skeletal anomalies involving the limbs or trunk were reported in 16 individuals (16/42), most notably congenital limb malformations, scoliosis and joint laxity. Of note, our patient’s elbow joint contracture represents a clinical feature not previously documented in *CLTC*-related literature.

Other rare systemic involvements included urogenital abnormalities (8/42), sleep disturbances (5/42), hearing deficits (4/42), hematological abnormalities (2/42) and other birth defects (7/42). Non-specific complications, such as hyperbilirubinemia, hypoglycemia, intraventricular hemorrhage, and recurrent ENT infections, were also noted in a small subset of cases.

### 3.4. Genotype of CLTC-Related Disorder Patients

**Figure 2:**
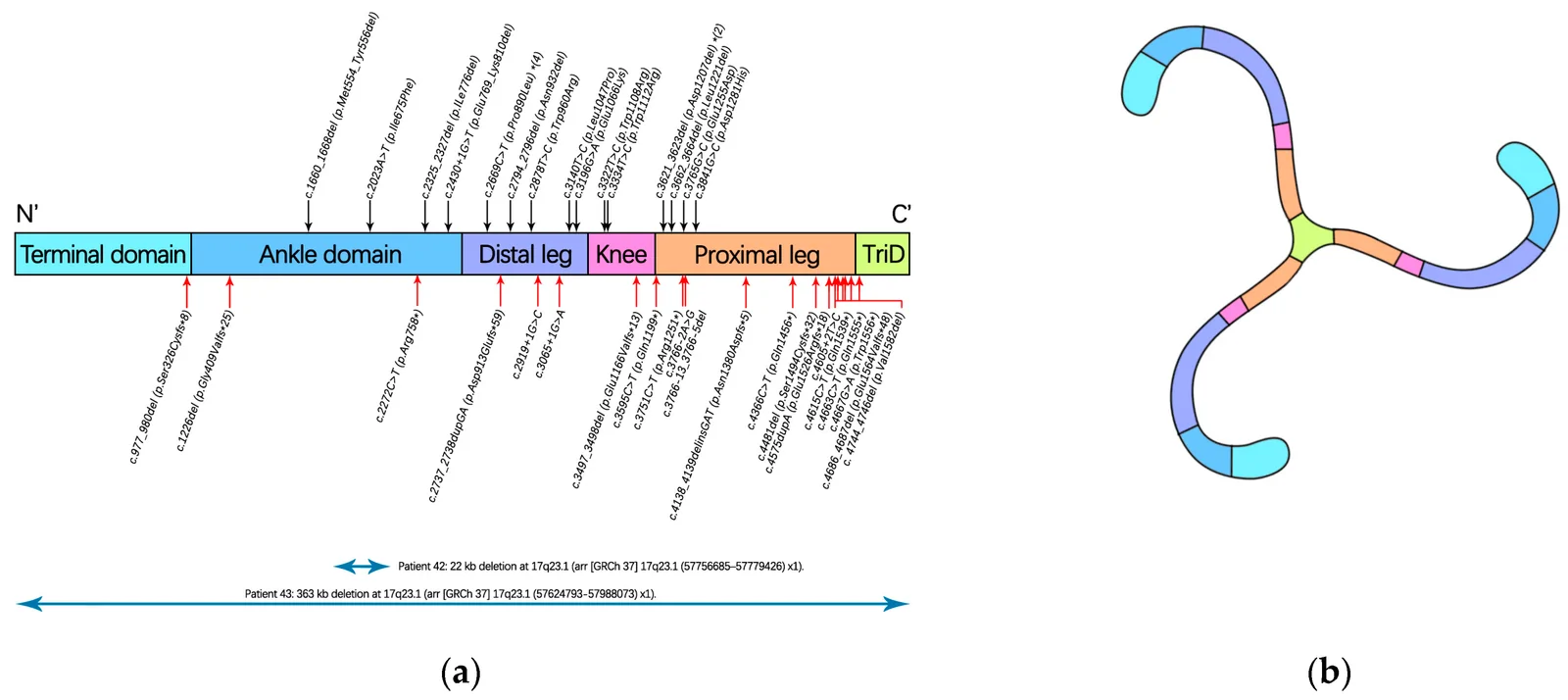
Schematic presentation of *CLTC* variants: (**a**) distinct *CLTC* variants documented in patients; (**b**) the structure of *CLTC*.

To date, 36 distinct *CLTC* variants have been documented in 40 patients, comprising nine missense variants, six in-frame deletions, eight frameshift variants, five splice-site variants, seven nonsense variants, and one mosaic in-frame deletion (c.4744_4746del) (Figure 2, Table 1 and Table S1). The c.2669C>T variant was the most frequent that has been observed in four patients, while the c.3621_3623del was identified in two patients. In addition to these small-scale variants, one larger 22.7-kb deletion (chr17:57756685–57779426), spanning exon 18 to the transcription end of *CLTC*, has also been reported. Moreover, Patient 42 represents the first complete *CLTC* gene deletion. Approximately 90% of these reported *CLTC* variants were de novo, reflecting the predominant genetic mechanism.

Notably, two patients in our analysis harbored co-occurring variants in other genes. Patient 8 carried a missense variant in *DNMT3A* and presented with ID, hypotonia, feeding difficulties, facial dysmorphism, nystagmus, ganglioneuroblastoma, and T-cell lymphoblastic lymphoma [11,16]. Given the established oncogenic role of *DNMT3A*, the tumoral manifestations in this patient were likely attributable to *DNMT3A* rather than *CLTC*. Patient 41 exhibited a 17q23.1 deletion encompassing both *CLTC* and *PTRH2*, consistent with our patient’s genetic findings. As *PTRH2*-related disease is autosomal recessive, its contribution to the phenotype is improbable. The multisystem manifestations in Patient 41 further support *CLTC* haploinsufficiency as the primary pathogenic driver.

## 4. Discussion

In this study, we reported the first documented case of a complete *CLTC* gene deletion. Although the patient exhibited hallmark neurodevelopmental features including GDD, ID, and emotional instability, congenital nystagmus was among the most easily identifiable clinical manifestations for differential diagnosis in the early postnatal period. Brain MRI performed in early infancy revealed mild hydrocephalus, although concurrent ophthalmologic evaluations demonstrated no structural ocular defects. Notably, the nystagmus persisted despite spontaneous resolution of the hydrocephalus, suggesting an intrinsic rather than secondary etiology.

Genetic analysis identified a *de novo* 363-kb heterozygous deletion at 17q23.1 [GRCh37: chr17:57,624,793-57,988,073], encompassing *CLTC*, *PTRH2*, *DHX40*, *VMP1*, *MIR21*, and *TUBD1*. To identify the primary driver of the phenotype within this region, we evaluated the haploinsufficiency constraint of each gene using the gnomAD. *PTRH2*, *DHX40*, and *TUBD1* are all haploinsufficiency-tolerant (pLI≈0), with heterozygous deletions frequently observed in the general population without disease association, thus excluding them as candidate genes. *MIR21*, a non-coding RNA primarily implicated in oncogenesis [27,28], has not been associated with congenital ocular abnormalities. Within the deleted region, only *CLTC* and *VMP1* exhibited haploinsufficiency intolerance (pLI≈1.00). Although *VMP1* shows weak expression in the retina [29], there is insufficient evidence linking it to ocular structural defects. In contrast, *CLTC* haploinsufficiency has been established as causative for developmental delay and nystagmus [11], demonstrating strong phenotypic concordance. These findings strongly implicate *CLTC* as the primary disease-causing gene.

The *CLTC* gene encodes CHC, comprising seven functional domains: the trimerization domain (TxD), proximal leg, knee, distal leg, ankle, linker, and terminal domain (TD) [30,31]. Mapping 38 reported coding variants onto this architecture revealed marked clustering in the proximal and distal leg segments (17 and 8 variants, respectively), collectively accounting for 65.8% (25/38) of all variants, with the remainder distributed across the ankle (*n* = 6), knee (*n* = 3), linker (*n* = 2), TxD (*n* = 1), and TD (*n* = 1) (Figure 2). These domains mediate protein–protein interactions essential for clathrin lattice assembly and cargo recognition in vitro [30,32], consistent with their enrichment for pathogenic variants.

Among the 42 patients, a genotype-driven severity gradient was observed. 11 patients exhibited severe GDD and/or ID carried predominantly missense variants (*n* = 6) and in-frame deletions (*n* = 4), whereas only one severe patient with nonsense variants exhibited moderate-to-severe ID. Missense variants and in-frame deletions may exert dominant-negative effects, as has been proposed for other neurodevelopmental disorders [33,34]. Aberrant proteins might incorporate into clathrin lattices and disrupt assembly or cargo trafficking, potentially impairing endocytosis more profoundly than haploinsufficiency alone. This hypothesis could explain the severe phenotypes associated with missense variants, although functional validation in *CLTC* is lacking. In contrast, truncating variants may undergo nonsense-mediated decay, preserving partial wild-type function through the residual allele, consistent with the milder phenotype observed in our patient with complete gene deletion.

Based on our literature review, nystagmus has been documented in three cases (Patients 8, 30, and 38) harboring *CLTC* variants c.2737_2738dupGA (p.Asp913Glufs*59), c.2878T>C (p.Trp960Arg), and c.3765G>C (p.Glu1255Asp), with onset ages of 3.5 year, 6 h after birth, and unspecified, respectively. Since no other deleted genes in our patient have been linked to ocular symptoms, we propose that the congenital nystagmus in this case is attributable to *CLTC* haploinsufficiency.

Our findings highlight congenital nystagmus as a potential early diagnostic indicator for *CLTC*-related disorder. In this cohort, ocular abnormalities were present in 39.5% (17/42) of patients, with nystagmus documented in at least three cases with diverse variant types and structural domain locations. This suggests that ocular involvement is a general feature of *CLTC* dysfunction rather than a domain-specific effect. *CLTC* mRNA is expressed in the retina, retinal pigment epithelium, and optic nerve [35], supporting a functional role in visual development. The early onset of nystagmus often precedes formal developmental assessment, underscoring its value as a clinical red flag. In the absence of overt craniofacial dysmorphism, congenital nystagmus accompanied by hypotonia or developmental delay should prompt clinicians to include *CLTC* in the differential diagnostic workup.

The broad phenotypic spectrum of *CLTC*-related disorder necessitates a structured diagnostic and management approach. We recommend: (i) detailed neurodevelopmental and ophthalmologic evaluation at initial presentation; (ii) brain MRI to exclude structural causes of nystagmus; and (iii) chromosomal microarray and/or trio exome sequencing with specific attention to *CLTC* when ocular findings co-occur with neurodevelopmental delay. Early genetic confirmation facilitates multidisciplinary management, informed genetic counseling, and surveillance for multisystem involvement. Beyond the core neurological features, clinicians should maintain vigilance for gastrointestinal dysfunction (42.86%), urogenital abnormalities (19.05%), and hearing loss (9.52%), which may manifest subclinically or progress over time.

To operationalize this approach, we developed a clinical management checklist for systematic care of patients with *CLTC*-related disorder (Table 2), encompassing developmental, neurological, ophthalmologic, gastrointestinal, urogenital, and auditory surveillance domains. This framework aims to standardize care and enable timely intervention across all potentially affected organ systems.

Several limitations of this study should be acknowledged. First, *CLTC*-related disorder is an ultra-rare condition, with only 42 affected individuals reported to date; therefore, the relatively small number of available cases limits the ability to establish definitive genotype–phenotype correlations or determine the true prevalence of individual clinical features. Second, our literature review relies primarily on previously published case reports and small case series, in which the extent of clinical evaluation and phenotypic documentation varied considerably. Consequently, the absence of a specific feature in a published case does not necessarily indicate that the feature was truly absent, introducing the possibility of ascertainment and reporting bias. Third, although our patient carries a *de novo* whole-gene *CLTC* deletion, this finding primarily supports the previously established haploinsufficiency mechanism rather than identifying a novel molecular mechanism. Finally, observations regarding potentially underrecognized manifestations, including early-onset nystagmus and musculoskeletal findings, are based on a limited number of patients and should therefore be interpreted cautiously. Additional patients with detailed prospectively longitudinal phenotyping and molecular characterization will be necessary to validate these associations and further define the natural history and phenotypic spectrum of *CLTC*-related disorder.

## 5. Conclusions

*CLTC*-related disorders are characterized by a broad phenotypic spectrum, with GDD/ID as predominant features. Systemic involvement frequently includes seizures, abnormal muscle tone, facial dysmorphism, gastrointestinal dysfunction, and ocular abnormalities. Given this clinical heterogeneity, a comprehensive multidisciplinary evaluation and consistent long-term surveillance are essential for optimal management.

Genotype–phenotype correlations remain to be elucidated. Our analysis suggests that missense variants and in-frame deletions are associated with more severe presentations than truncating variants. Additionally, clustering of variants within the proximal and distal leg domains of CHC implicates these regions as mutational hotspots. These findings warrant further functional studies to clarify how domain-specific alterations disrupt protein function and modulate clinical severity.

## Figures and Tables

**Table 1:** Genetic features of 42 patients with *CLTC* variants.

Characteristic	
*Demographics*	
Male	20/42
Female	22/42
Age at assessment	3 mo–41 yr
*Genetics*	
*De novo* vs. inherited vs. unknown	37 vs. 4 vs. 1
Missense variant (patients/variants)	12/9
In-frame deletion (patients/variants)	7/6
Frameshift variant (patients/variants)	8/8
Splice-site variant (patients/variants)	5/5
Nonsense variant (patients/variants)	7/7
Mosaic in-frame deletion (patients/variants)	1/1
Copy number variant (patients/variants)	2/2

**Table 2:** Recommendations for management and surveillance of patients with *CLTC*-related disorder.

Specialty	Recommendations
Development (Developmental delay/ Intellectual disability)	Monitor development and learning ability, provide early intervention (ST, OT, PT) and Individualized Education Program (IEP) as indicated.
Neurology	Refer to neurology if clinical concerns for seizure, abnormal muscle tone, abnormal gait or movement, consider imaging study as indicated. Evaluation for autism spectrum disorder and ADHD if clinically concerned.
Feeding difficulty/Gastrointestinal abnormalities	Evaluate nutrition and growth at every visit. Implement feeding therapy as needed to improve feeding difficulty or dysphagia. Monitoring and treatment for GERD and constipation.
Ophthalmologic abnormalities	Refer at diagnosis to screen for strabismus, nystagmus, visual impairment, refractive errors, abnormal eye movements, etc.
Hearing loss	Hearing screening at diagnosis.
Sleeping disturbance	Obtain a detailed sleep history. Manage sleep disorders through a multifaceted approach combining tailored sleep hygiene interventions and pharmaceutical agents.
Other anomalies	Perform renal ultrasound to evaluate for kidney abnormalities and/or echocardiography to assess for cardiac anomalies as clinically indicated.

## Data Availability

Data are available upon reasonable request.

## Supplementary Materials

The following supporting information can be downloaded at: https://www.mdpi.com/article/10.3390/genes17080928/s1. Table S1: Genotype and Phenotype of *CLTC*.

## Abbreviations

The following abbreviations are used in this manuscript:

ASD	Autism spectrum disorder
BMI	Body mass index
CHC	Clathrin heavy chains
CMA	Chromosomal microarray analysis
DD	Developmental delay
GDD	Global developmental delay
ID	Intellectual disability
TD	Terminal domain
TxD	Trimerization domain
